# Efficient treatment of chronic endometritis through a novel approach of intrauterine antibiotic infusion: a case series

**DOI:** 10.1186/s12905-018-0688-8

**Published:** 2018-12-05

**Authors:** Konstantinos Sfakianoudis, Mara Simopoulou, Yorgos Nikas, Anna Rapani, Nikolaos Nitsos, Katerina Pierouli, Athanasios Pappas, Agni Pantou, Christina Markomichali, Michael Koutsilieris, Konstantinos Pantos

**Affiliations:** 1 Centre for Human Reproduction, Genesis Athens Clinic, 14-16, Papanikoli 15232, Athens, Greece; 2 Department of Physiology, Medical School, National and Kapodistrian University of Athens, 75, Mikras Asias, 11527 Athens, Greece; 3 Assisted Conception Unit, 2nd Department of Obstetrics and Gynecology, Aretaieion Hospital, Medical School, National and Kapodistrian University of Athens, Athens, Greece; 4 Athens Innovative Microscopy, 36, Skra, 16673 Voula, Athens Greece; 5 Microbiology-Biochemical Department, Genesis Athens Clinic, 14-16, Papanikoli 15232, Athens, Greece

**Keywords:** Chronic endometritis, Antibiotic, Intrauterine infusion, Natural conception

## Abstract

**Background:**

Early diagnosis and efficient management of Chronic Endometritis (CE) in patients seeking fertility treatment are two components every practitioner wishes to address. With respect to endometrial restoration, antibiotic treatment appears to perform well. However, regarding the improvement of In Vitro Fertilization (IVF) success rates, literature evidence is inconclusive, and consensus on optimal treatment has yet to be reached. This manuscript uniquely brings to literature the first report on effective employment of intrauterine antibiotic infusion to treat CE and contribute to addressing the infertility related to it.

**Case presentation:**

In this case series, we present 3 patients reporting numerous previous failed IVF attempts accompanied with diagnosed CE which failed to be properly treated in the past. Following initial assessment in our clinic and verification of CE findings, an oral antibiotic regime was administered based on the infectious agent detected. Re-evaluation concluded slightly improved microbiological environment in the endometrium but persisting inflammation. Antibiotic intrauterine infusion was proposed to the patients as an alternative practice. All our patients achieved a pregnancy shortly following intrauterine treatment with one patient reporting a live birth of twin babies and two patients currently reporting an ongoing pregnancy.

**Conclusions:**

The implications of this case series contribute to medical knowledge and extend to both effective treatment of CE and subsequent management of related infertility. The current line of treatment of CE through oral antibiotic regimes highlights the need for exploring new options and calls for larger studies on the clinical implication of their use. This novel approach enabled natural conception for patients presenting with established Recurrent Implantation Failure (RIF) having undergone numerous futile IVF attempts. The clinical impact from the practitioner’s perspective is considerable allowing for an alternative line of treatment that merits further investigation.

## Background

Chronic endometritis (CE) is a serious medical condition implicated in 12–46% cases of infertile patients [1]. Inflammation of the endometrium is the basic feature described in this condition accompanied with the presence of a heightened percentage of plasma cells along with several infectious agents associated with the symptomatology of the disease [2]. Embracing the idea that CE and infertility share a strong connection was not until recently introduced as an area of interest.

The complex nature related to its diagnosis and treatment renders CE a time-consuming matter to address. A method primarily implemented for diagnosis is the histological examination of the endometrial biopsy, which is considered to be the golden standard diagnostic tool in the hands of practitioners nowadays [3]. However, due to the presence of inflammatory cells in the endometrium under normal circumstances, diagnosis of CE is rendered challenging. The employment of fluid hysteroscopy may assist further through the reveal of micro polyps, stromal edema and focal or diffuse hyperemia [4]. The association of CE with the presence of eosinophils in the endometrium along with the employment of antibody CD138 for plasma cells have emerged with a promise of a more effective diagnosis [5]. Established criteria for diagnosis are required in order to ascertain optimal diagnosis and a timely and successful management.

In particular, during In Vitro Fertilization (IVF) treatment, CE is expected to be encountered in about 42% of women who present with Recurrent Implantation Failure (RIF) [6]. Endometrial receptivity -a factor exerting enormous impact on an IVF cycle- is jeopardized by the abnormal endometrial microenvironment characterizing CE [7]. Patients reporting chronic endometritis accompanied with RIF present poor success rates in IVF cycles. The subtle pathology of CE renders diagnosis a difficult task to perform [3], especially in IVF patients for whom time is of the essence. Ultimately, practitioners opt for multiple diagnostic approaches in order to secure an accurate interpretation of the symptoms.

Treatment of CE relies on administration of various antibiotics depending on the type of the microbe that has been detected. The antibiotic treatments are primarily administered orally and endometrial re-examination is performed following treatment. It should be emphasized that apart from the differences in the types of antibiotics included, there are also significant discrepancies regarding the dosage of each antibiotic and the different schemes suggested by each practice. Following the antibiotic regime, endometrial receptivity was anticipated to improve. However, no significant correlation between antibiotic therapeutic approach and positive results in IVF has been stated in literature leaning towards the consensus that oral antibiotic administration fails to improve the IVF outcome [8, 9].

The immune responses presenting in CE rarely develop into systematic inflammation [10], thus the question raised by our team of experts was “why then apply a systematic antibiotic therapeutic protocol?” Hereby, we present an array of CE patients treated with intrauterine infusion of an antibiotic regime following a standard unsuccessful oral antibiotic scheme. The aim of this work focuses on presenting practitioners with an alternative novel approach relying principally on intrauterine antibiotic infusion for these patients and finds strength through combining present options into a more robust, reliable, more effective mode of successful management of CE.

### Case presentation

**Fig. 1:**
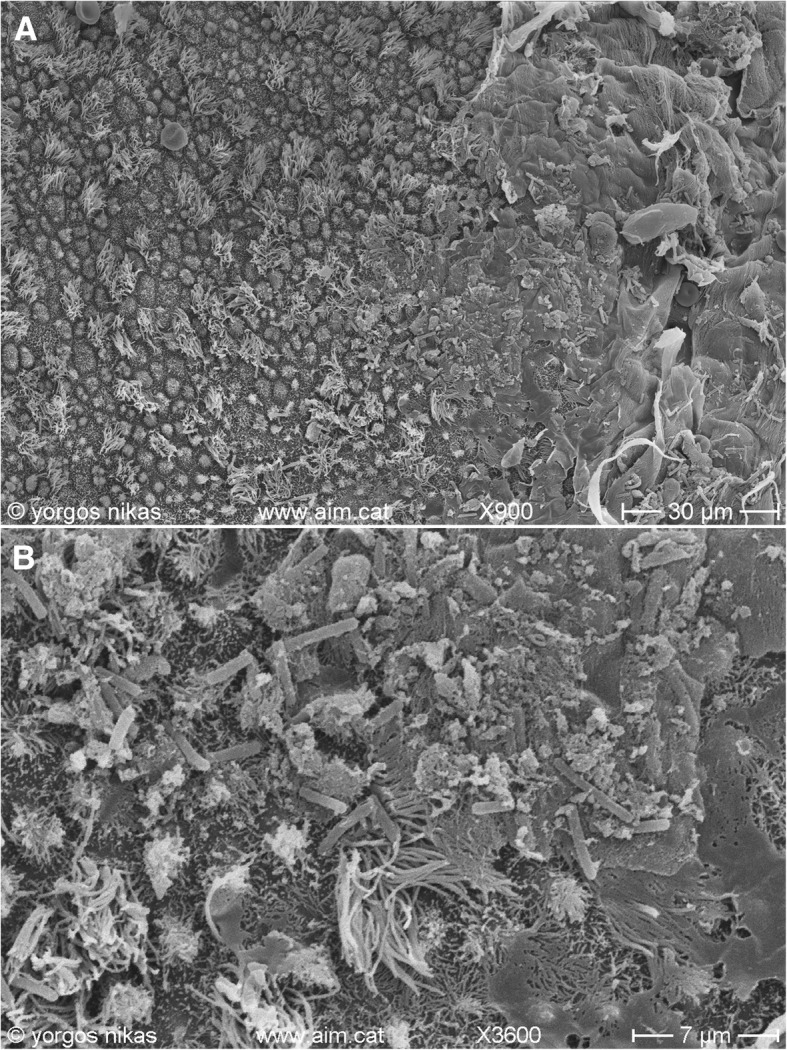
**a** SEM micrograph of endometrial surface from a patient with CE. Note the presence of thick mucus layer (right) containing debris and red blood cells. **b** Detail of Fig. 1a. Mucus and abundant bacteria are sticking on the microvilli and the cilia of the endometrial epithelial cells

All patients were referred to our Clinic reporting with numerous failed IVF attempts in the past and a diagnosis of CE. Investigation was performed on the grounds of RIF for all. Tubal patency was established for all patients. Semen analysis of the partners categorized the patients as non-male factor. Finally, the hormonal profile on Follicle-Stimulating Hormone (FSH), Luteinizing hormone (LH) and Anti-Müllerian Hormone (AMH) provided the required criteria for pursuing the possibility of natural conception. In particular, levels of FSH for the three patients were recorded respectively as follows Patient 1: 5.12 mIU/ml, Patient 2: 3.1 mIU/ml, Patient 3: 5.81 mIU/ml. Additionally, AMH levels were recorded as follows Patient 1: 18 ng/ml, Patient 2: 26 ng/ml, Patient 3: 20 ng/ml. The overall healthy fertility status that was concluded for all was indeed subverted by the presence of CE, as histological examination of the endometrial biopsy and microbiological analysis provided evidence of endometritis. This was further depicted in Fig. 1. The following section reports on the management of these patients, the approach being identical for all three. Thorough information is provided regarding the protocols employed on CE management for diagnosis, therapy and addressing infertility.

### Diagnostic hysteroscopy

All our patients underwent diagnostic hysteroscopy at the follicular phase of the menstrual cycle, using a lens-based 3 mm OD mini-telescope, 1058 angle of visual field equipped with a 3.5 mm OD single-flow diagnostic sheath. Saline was employed to distend the uterine cavity, while a 300 W light source with a xenon bulb, a digital camera and a 21 in. video colour screen were used. A panoramic view of the cavity and a thorough evaluation of the endometrial mucosa were performed. The diagnosis of endometritis was made in all cases taking into account the presence of polypoid endometrium, micropolyps, stromal edema and diffuse hyperaemia.

### Endometrial biopsy

An endometrial biopsy using a 3 mm Novak’s curette connected to a 20 ml syringe was performed for the infectious agent analysis. In order to minimize the contamination risk, after placing a vaginal speculum and cleaning external uterine ostium with an iodine solution, the Novak’s cannula was inserted under visual control into the uterine cavity taking care to avoid any contact with vaginal walls. Endometrial samples were diluted into 2 ml of saline and divided into two aliquots, for histological and microbiological analyses.

### Histological analysis

For the fixation of the endometrium samples, neutral formalin and paraffin were employed. In order to stain the microsections, hematoxyline and eosin were engaged. The presence of increased stromal density, superficial stromal edema and pleo-morphic stromal inflammation were the main findings consisting evidence of CE presence.

### Microbiological analysis

Endometrial samples were Gram stained and further placed to agar medium, 5% sheep blood Columbia Agar Base, Chocolate Agar, Mannitol Salt Agar and Mac Conkey Agar (Bio Merieux, Rome, Italy). The samples were incubated for 48 h in air or 5% CO_2_ prior to evaluation. For the identification of bacteria, published criteria were employed (Dade International Inc., Milan, Italy). The analysis revealed *Mycoplasma species* and *Ureaplasma urealyticum* agents that are being described as the most frequent etiologic factor in literature.

### Oral antibiotic regime

Based on the infectious agents detected and on the antibiogram result, an appropriate antibiotic treatment was administered. Following guidelines and clinical practice routine set by our clinic, the regime included doxycycline. This orally administered antibiotic was prescribed for a period of 21 days.

### Re-evaluation and intrauterine antibiotic infusion treatment

**Fig. 2:**
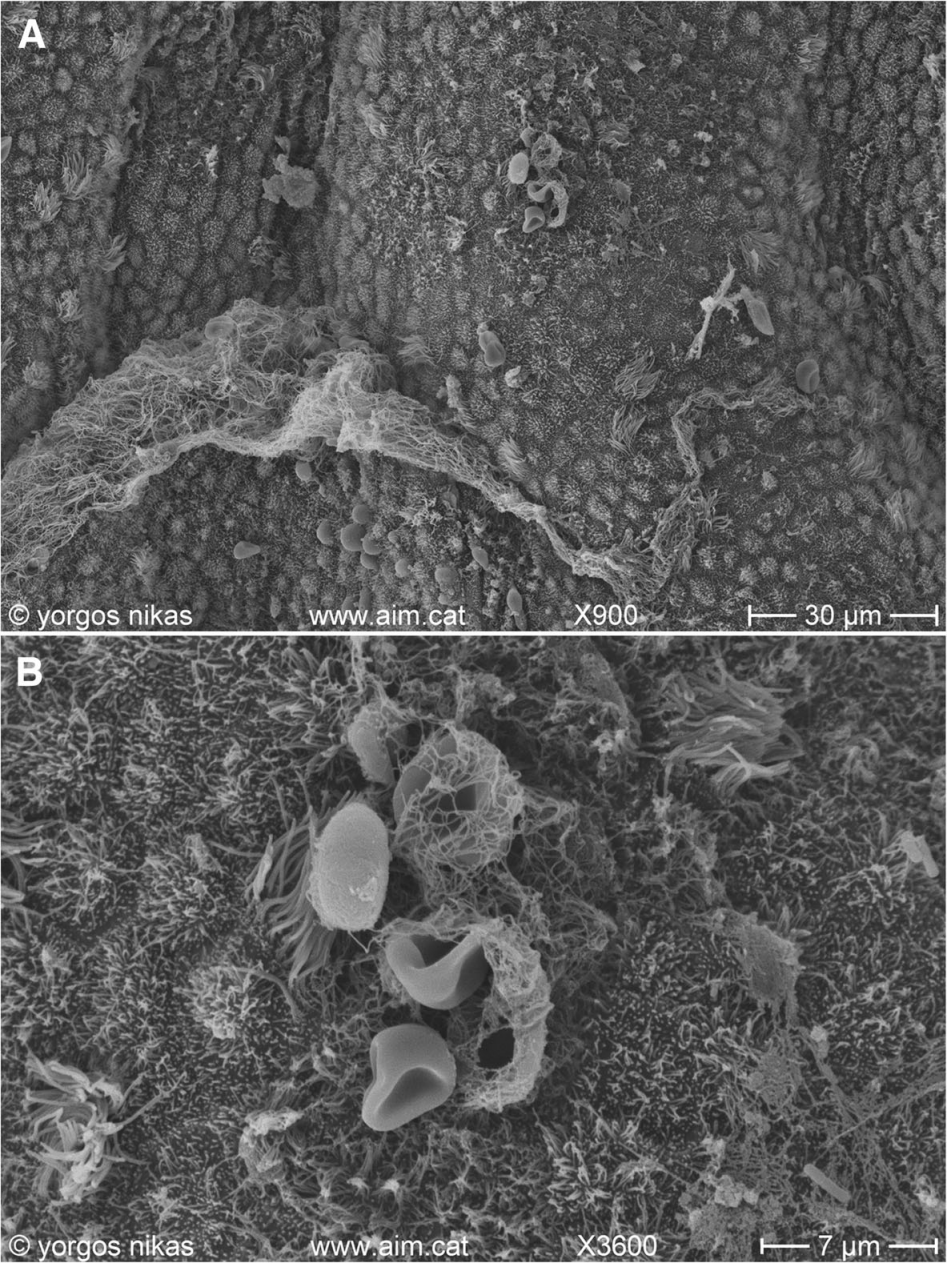
**a** Endometrial surface after oral treatment for CE. Note the presence of filamentous mucus (lower) and aggregations of red blood cells (upper middle). **b** Detail of Fig. 2a. Fibrinous material with red blood cells (middle) -possibly emerging through diapedesis- and isolated bacteria (middle and lower right) are seen on the epithelium

Following traditional treatment of CE employing oral antibiotic administration, histological and microbiological re-evaluation provided evidence of a partially improved condition -regarding the severity of CE. Nonetheless, it indicated failure in significantly treating CE as revaluation results were overall rather discouraging. In all three cases following oral antibiotic administration, the condition of the endometrial environment was described as slightly improved but persisting, as histological and microbiological findings indicated the persistence of inflammation and infectious agents in all three patients. Scanning Electron Microscope (SEM) evaluation depicted the improved but persisting condition (Fig. 2).

In an effort to provide an enhanced efficiency approach in treating the persistent nature of CE, patients were offered the option of intrauterine antibiotic infusion. All patients provided written informed consent prior to embarking on intrauterine antibiotic infusion treatment. The Ethics Board of Genesis Athens Clinic approved the study protocol in accordance to the Helsinki declaration.

Details on the strategy design regarding the therapeutic protocol follow. According to the administration guidelines of ciproxin as outlined in the supplementary protection certificate of the antibiotic agent, it is recommended that antibiotic treatment in patients reporting with pelvic inflammatory disease (PID) should consist of 10 treatment days. Based on that, our treatment similarly included 10 treatment days respectively. Nonetheless, intrauterine infusion treatment could not be performed in 10 consecutive days of treatment. Each intrauterine infusion was performed every 3 days for the reason that fluid from the previous infusion was still present in the cavity when observed on the second day following administration, an observation that was cleared on the third day. Therefore, the treatment cycle was set for a period of one month including 10 infusions within this time frame. With regards to the volume employed per intrauterine infusion, each infusion included 3-4 ml, a volume that is consistent with the cavity’s maximum capacity. Regarding the antibiotic regime employed, our protocol included the solution for intravenous infusion of ciprofloxacin at a concentration of 200 mg/100 ml. Antibiotic infusion was performed employing a 23 cm soft Embryo Replacement Catheter.

**Fig. 3:**
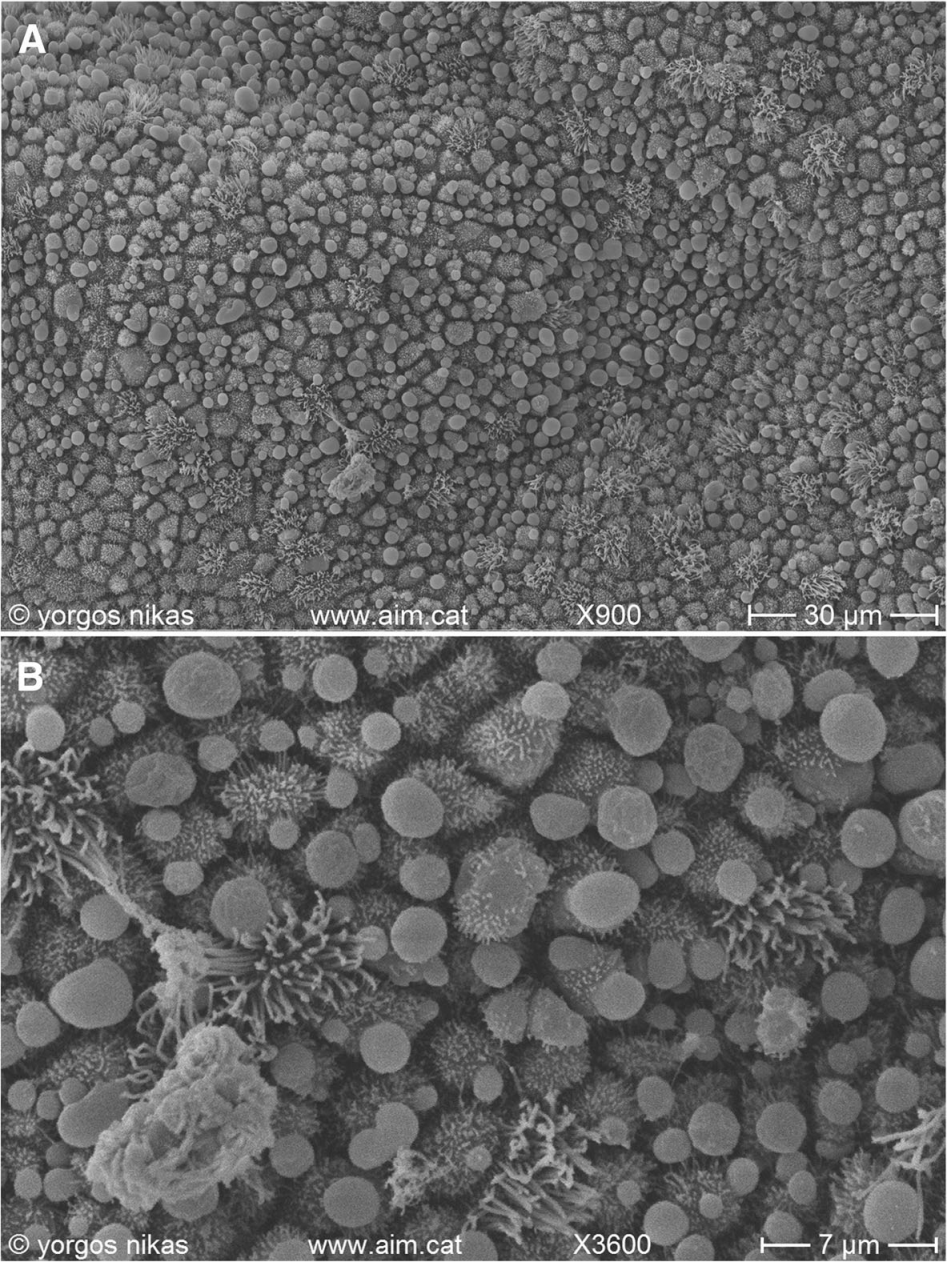
**a** Endometrial surface after intrauterine treatment for CE. The surface appears clean with abundant pinopodes. **b** Detail of Fig. 3a. Note the presence of fully developed pinopodes and of a small mucus aggregate (lower left)

Following treatment, patients were referred for a re-evaluation during the subsequent menstrual cycle follicular phase with endometrial biopsy, for histological and microbiological examination -as previously described. The reassessment provided encouraging evidence that intrauterine antibiotic infusion treatment was not only well tolerated by the patients without any adverse or unanticipated events occurring, but also it managed to majorly mitigate the signs of endometritis. This was further depicted in Fig. 3. Establishing efficiency of the treatment and describing good physiology and condition of the endometrium patients were then invited to pursue pregnancy via natural conception. Natural conceptions were ensued for all 3 patients. Patient 1 reported a spontaneous conception 4 months following intrauterine infusion. Patient 2 naturally conceived in 2 months and Patient 3 in 6 months following treatment. Patient 1 at 35 years old presented with a history of 6 previous failed IVF attempts and 7 years of infertility. Patient 2 at the age of 38 presented with 3 previous failed IVF attempts and 5 years of infertility. Patient 3 at the age of 33 was accompanied with 4 previous failed IVF attempts and 4 years of infertility. Both patients 1 and 3 report ongoing complication-free pregnancies at 19 weeks and 20 weeks accordingly. For patient 2, natural conception following intrauterine treatment of CE resulted to the birth of twin babies at the 37th week of gestation. Their respective weight was 2660 g and 2680 g with excellent APGAR scores.

Referral reason for all patients was focused on RIF and CE. The severity of RIF described by failed IVF attempts ranged from 3 to 6 cycles of unsuccessful IVF treatment and years of infertility ranged from 4 to 7. All patients aged 33–38 were subjected to an identical protocol of treatment including an oral antibiotic approach followed by a first evaluation and subsequent intrauterine infusion on the grounds of unsuccessful treatment followed by a final evaluation confirming effective management. It is worth noting that the age and burdened reproductive history were the only distinguishing elements in this case series.

All hysteroscopies were performed by KS. SEM analyses were performed at the Centre Athens Innovative Microscopy (AIM) and respective SEM images were developed by and provided by YN. All microbiological analyses were performed by NN and all intrauterine antibiotic infusions were performed by KS.

## Discussion and conclusions

Unsatisfactory results and numerous reports on the inconsistency of CE treatment clearly indicate that present options lack the desired efficiency that is required in order to manage successfully this challenging condition which is extending to affect fertility status. The search for optimal treatment of CE is still ongoing on various levels: from administration, to strength of antibiotic regime, to duration of treatment and time, and method of assessing effectiveness. Practitioners may be faced with confusion regarding the optimal approach as current data is confounding. Adding infertility and IVF failure into the equation adds another level of complexity and highlights the requirement for efficient treatment ascertaining optimal results in a timely fashion. The element of time should be considered and examined separately as patients addressing infertility issues are best managed when treated within a time sensitive frame. In addition to that, CE patients receiving fertility treatment may be faced with a further underlying infertility etiology complicating treatment and resulting to delayed diagnosis.

Unable to be properly diagnosed in a timely manner, CE may become the underlying factor leading to IVF overuse for a respected percentage of patients aiming to conceive. Continuous failed IVF attempts associated with CE may result to frustration, stress, psychological and financial uncertainly and subsequently higher risk of complications for the patients [11, 12]. In most of the cases, this overuse will ultimately fail to provide the desirable outcome of a conception, since the underlying mechanism of infertility remains either undetected or untreated. The implication of chronic endometritis in infertility issues has been proposed to include the possible effect of microbes on the endometrial receptivity [13]. More precisely, lymphocyte subsets and an overall abnormal micro-environment may hamper endometrial receptivity [7] which may be subsequently reflected as the embryo’s failure to implant for numerous cases. The mechanism orchestrating this fails to be entirely deciphered. However, it is suggested that the presence of microbes in the uterine cavity constitutes the trigger mechanism of immune responses. Those responses result to the aforementioned abnormal micro-environment for the recruitment of circulating B cells, along with elevated levels of numerous factors associated with inflammation [14]. Moreover, findings propose that a pathological endometrium affected by CE fails to respond to ovarian steroids that are commonly employed in order to render the endometrium receptive [14].

It has been proposed in cattle that cases of uterine bacterial contamination are directly associated with the aggravation of findings such as the disruption of the epithelium’s integrity, coupled by an influx of neutrophils and secretion of chemokines and cytokines, drawing the scenery of a persistent inflammatory response [15]. Subsequently, endocrine disfunction is ensued in the endometrium, presumably disrupting the embryo’s implantation [16]. It is further documented that in bovine embryos, a reduced number of trophoctoderm cells is present when development has taken place in inflamed conditions [17].

Animal studies and application of intrauterine antibiotic infusion reporting with interesting results served as the basis for our approach [18]. Literature research resulted to multiple studies reporting discrepancies. It is already proposed that the intrauterine infusion on animal models could be beneficial [19] whereas it has also been accompanied with negative results [20]. In humans, oral antibiotics allegedly fail to treat all cases of CE, presenting with 10% failure rate [21]. Instead, local delivery of antibiotics has been introduced as a method of improved efficiency. However, in various fields of medicine, local employment of antibiotics may be coupled by hesitation on the practitioners’ perspective. The principal dilemma regarding local application revolves around the fact that following a local infusion, and in the case of development of a subsequent bacterial resistance then, performing a systematic infusion may present to be of futile efficiency [22]. On the contrary, it is also argued that local infusion may be associated with low levels of systematic absorption along with mitigated side effects enriching its portfolio [23]. Performing local antibiotic infusion in our patients was a clinical insight based on literature evidence supporting that the delivery of local antibiotics could potentially replace the implementation of systematic antibiotics [24]. This allows for high antibiotic schemes’ concentrations administered locally circumventing the risk of systemic toxicity. Further to that, resistant bacteria that may be developed in response to regular antibiotic concentrations may in fact be manageable employing an extremely high concentration administered locally [24]. Both benefits related to localized administration as described above served adequately as the basis of this novel approach.

Regarding the underlying mechanism elucidating the role of intrauterine infused antibiotics, an animal study in dairy cows suggests the manifestation of various events. The presence of pathogens in the uterine cavity is alleviated, the findings of inflammation are subverted, the local immune defense is enhanced, resulting to the restoration of the endometrium [25]. We could hypothetically extrapolate that the aforementioned rationale could be extended to humans.

The failure of the first oral antibiotic regime was evident by subsequent microbiological and histological investigation concurring on the slightly improved but nonetheless persisting CE. SEM further confirmed that CE had been insignificantly treated hitherto. Our 3 patients presented with a notable psychological frustration and a limited time frame for treatment following their numerous previous failed attempts, while taking into account their respective age. Nonetheless, these patients persisted in pursuing fertility treatment prompting our team of specialists to explore alternative means of treatment to circumvent the challenge of efficient management and ascertainment of a positive result. Localized administration of antibiotics in the form of intrauterine infusion surfaced as a valid option based on published data described above. The authors cannot claim that this novel approach was buttressed only by reassuring data regarding safety, as respective animal studies reporting on efficiency and safety may be viewed as contradictory [19, 20]. Therefore, the authors proceeded with an informed decision and chose to base this new line of treatment for this case series relying on the adequate published animal models data presenting intrauterine administration of antibiotics as an efficient and safe option. Further to that, it is of essence to highlight that the practitioners decided that the management of these patients regarding CE treatment reached a plateau. Such an acknowledgment would fuel further futile IVF attempts with the risk of IVF overuse and all that this entails, or would lead towards the exploration of the option of embarking on a surrogate program addressing a compromised unreceptive endometrium.

Prior to concurring on further treatment, the possible safety issues raised were thoroughly discussed. The risk of promoting resistant strains as the principal safety issue identified was concurred on as being of equal weight, if not conveying a lowered risk in comparison to the alternative option of proceeding with the approach of repeating an oral administered treatment protocol for 3 times [26]. At that point regarding the next step, selection of the antibiotic agent was investigated. The risk of adhesions that may be encountered was alleviated as the antibiotic scheme of choice has not been associated with such complications in comparison to others. On the scale of evaluating the questionable prognosis option of repeated IVF attempts for RIF patients or surrogacy, the practitioners felt that embarking on a novel approach acknowledging the unknown territory would have more to offer.

Our results indicated that this novel approach improved significantly the overall endometrial environment of our patients -a phenomenon that orally administered antibiotics failed to accomplish in the first line of management. This extended to enabling natural conception in all patients following years of infertility and of futile IVF attempts. However, numerous questions are raised that our study is not arrayed to clarify. Randomized controlled trials are required to define the duration of the improved effect and whether it institutes a permanent condition, as well as to provide insight on regards to the periodicity, volume and the appropriate scheme of antibiotics. Further to that the perils associated with generating antibiotic resistant criteria should not be overlooked especially in cases of inadequate-yet repeated -treatment of chronic CE. Local antibiotic treatment is a common medical practice coupled with good results. Further to that, it may be extrapolated that in this case local administration could contribute towards mitigating the risks discussed in comparison to oral treatment. Providing evidence that the benefits outweigh the risks may establish this novel line of treatment.

Fine tuning of current treatments and evaluation on novel approaches applied on animal models may provide new possibilities that merit investigation. Our goal remains safe and effective practice. Therefore, it is imperative that proper examination of possible treatment scenarios presenting as promising should undergo meticulous evaluation ensuring validity along with legitimacy of a practice approved by the scientific community.

This case series cannot claim to provide adequate evidence to conclude on the employment of intrauterine infusion of antibiotic, but rather highlights the need for a change in clinical practice regarding CE treatment in the context of related infertility. Future validation of our findings in larger patient cohorts could cement this therapeutic approach as a beneficial and perhaps preferred approach to the treatment of patients with CE and contribute further to medical knowledge. This could be heightened especially in cases where conventional antibiotic schemes lead to time-consuming, futile attempts towards ineffective management of CE jeopardizing the patients’ reproductive options.

## Abbreviations

AIM: Centre athens innovative microscopy
AMH: Anti-Müllerian Hormone
CE: Chronic endometritis
FSH: Follicle-Stimulating Hormone
IVF: In Vitro Fertilization
LH: Luteinizing hormone
PID: Pelvic inflammatory disease
RIF: Recurrent implantation failure
SEM: Scanning Electron Microscope
